# Supplementary material for: Rare variant associations with plasma protein levels in the UK Biobank
Source: Nature. 2023 Oct 4;622(7982):339–47. doi: 10.1038/s41586-023-06547-x (PMC10567546; doi:10.1038/s41586-023-06547-x)
Supplement: Supplementary file 1 — Supplementary Note, Figs. 1–4, detailed descriptions of Datasets 1–20 and references. [file 41586_2023_6547_MOESM1_ESM.docx]

***Supplemental Information***

**Rare variant associations with plasma protein levels in the UK Biobank**

Ryan S. Dhindsa^1^*, Oliver S. Burren^2^*, Benjamin B. Sun^3^*, Bram P. Prins^2^, Dorota Matelska^2^, Eleanor Wheeler^2^, Jonathan Mitchell^2^, Erin Oerton^2^, Ventzislava A. Hristova^1^, Katherine R. Smith^2^, Keren Carss^2^, Sebastian Wasilewski^2^, Andrew R. Harper^4^, Dirk S. Paul^2^, Margarete A. Fabre^2^, Heiko Runz^3^, Coralie Viollet^2^, Benjamin Challis^5^, Adam Platt^6^, AstraZeneca Genomics Initiative, Dimitrios Vitsios^2^, Euan A. Ashley^7^, Christopher D. Whelan^3^, Menelas N. Pangalos^8^, Quanli Wang^1^, Slavé Petrovski^2,9^

^1^Centre for Genomics Research, Discovery Sciences, BioPharmaceuticals R&D, AstraZeneca, Gaithersburg, US

^2^Centre for Genomics Research, Discovery Sciences, BioPharmaceuticals R&D, AstraZeneca, Cambridge, UK

^3^Translational Biology, Research & Development, Biogen Inc., Cambridge, MA, US

^4^Clinical Development, Research and Early Development, Respiratory and Immunology (R&I), BioPharmaceuticals R&D, AstraZeneca, Cambridge, UK

^5^Translational Science and Experimental Medicine, Research and Early Development, Cardiovascular, Renal and Metabolism, BioPharmaceuticals R&D, AstraZeneca, Cambridge, UK

^6^Translational Science and Experimental Medicine, Research and Early Development, Respiratory and Immunology, BioPharmaceuticals R&D, AstraZeneca, Cambridge, UK

^7^Department of Medicine, Division of Cardiology, Stanford University, Palo Alto, CA, USA

^8^BioPharmaceuticals R&D, AstraZeneca, Cambridge, UK

^9^Department of Medicine, Austin Health, University of Melbourne, Melbourne, Australia

*These authors contributed equally

**Table of Contents**

[Supplementary Note 3](#_Toc140760825)

[Supplementary Figure 1 4](#_Toc140760826)

[Supplementary Figure 2 5](#_Toc140760827)

[Supplementary Figure 3 6](#_Toc140760828)

[Supplementary Figure 4 7](#_Toc140760829)

[Description of Supplementary Tables 8](#_Toc140760830)

[AstraZeneca Genomics Initiative banner contributors 11](#_Toc140760831)

[Supplementary References 13](#_Toc140760832)

## Supplementary Note

We sought to replicate our rare variant pQTLs in a separate cohort. Because most previous sequence-based rare variant proteogenomic studies had dramatically smaller sample sizes, we instead compared our pQTLs to a prior GWAS performed in an Icelandic population by Ferkingstad et al (2021).8 By using deep imputation with a large population-specific reference panel, they analyzed variants rare as MAF>0.01%. The authors used an additive model to perform a GWAS of plasma protein levels measured with 4,908 SomaLogic aptamers in 35,559 Icelanders.

Because the authors used an additive model, we aimed to compare results from our analogous ExWAS model (the “genotypic” model). Our genotypic model identified 5,435 rare pQTLS (MAF≤0.1% and p≤1x10^-8^). We matched these with the Ferkingstad summary statistics based on UniProt identifier, chromosome, position, and alleles. We were able to match 643 rare pQTLs (76.5% of which were *cis*-CDS signals), representing 337 unique proteins and 507 unique variants. In total, 53.18% of the UKB ExWAS pQTLs (342 out of 643) achieved nominal significance in Ferkingstad et al. (p<0.05). These replicated pQTLs spanned 203 unique proteins and 283 unique variants, and 79.5% were cis signals.

There was significant directional concordance between the replicated pQTLs, with 93.57% showing the same direction of effect (two-sided binomial test p = 5.99x10^-69^). The effect sizes were correlated with a Pearson’s R of 0.84 (p = 2.38x10^-91^). We consider this 53% replication rate quite strong given the differences in sequencing approaches (whole-exome sequencing vs. array and imputation), population (UK vs. Icelandic), sample sizes (46,327 vs. 35,559), and proteomic platforms (Olink vs. SomaLogic). A prior study showed that even within the same population and sequencing platform, only 64% of pQTLs were shared across Olink and SomaLogic.^2^ Another study suggested that Olink is more likely to measure the intended protein than SomaLogic.^3^

## Supplementary Figure 1

**
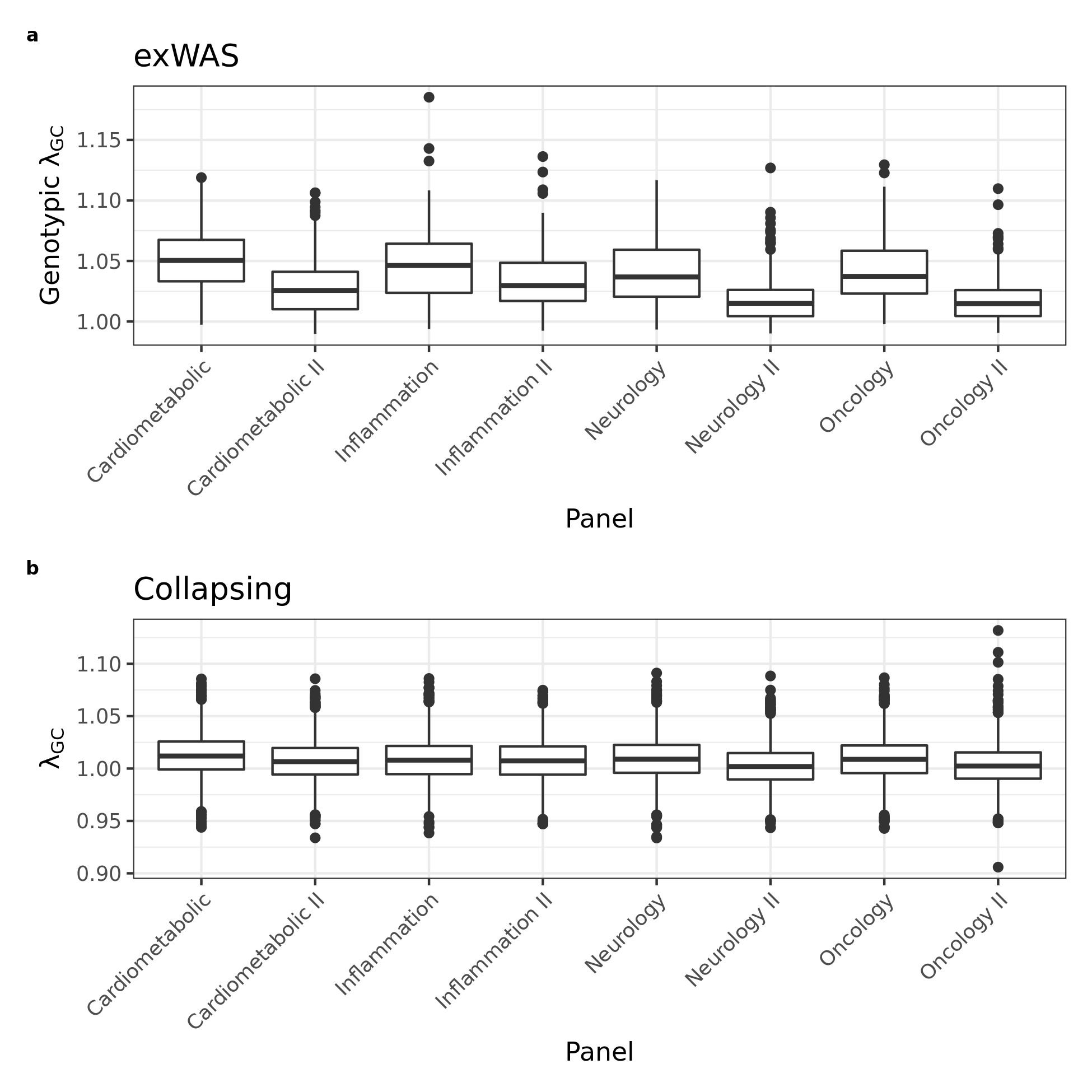
**

**Lambda genomic control boxplots by Olink panel.** Lambda_gc_ was computed using an n-of-1 permutation approach for **(a)** the ExWAS genotypic model and **(b)** the collapsing analysis. The boxplots show the median (centre line) and interquartile ranges (IQR) (box limits). The length of the whiskers corresponds to 1.5 × the IQR. The outlier values indicate those >1.5 times and <3 times the IQR beyond either end of the box. Cardiometabolic (p=369), Cardiometabolic II (p=367), Inflammation (p=368), Inflammation II (p=368), Neurology (p=367), Neurology II (p=366), Oncology (p=368), Oncology II (p=368), over n=46,327 biologically independent EUR ancestry individuals and “p” independent assays per panel.

## Supplementary Figure 2


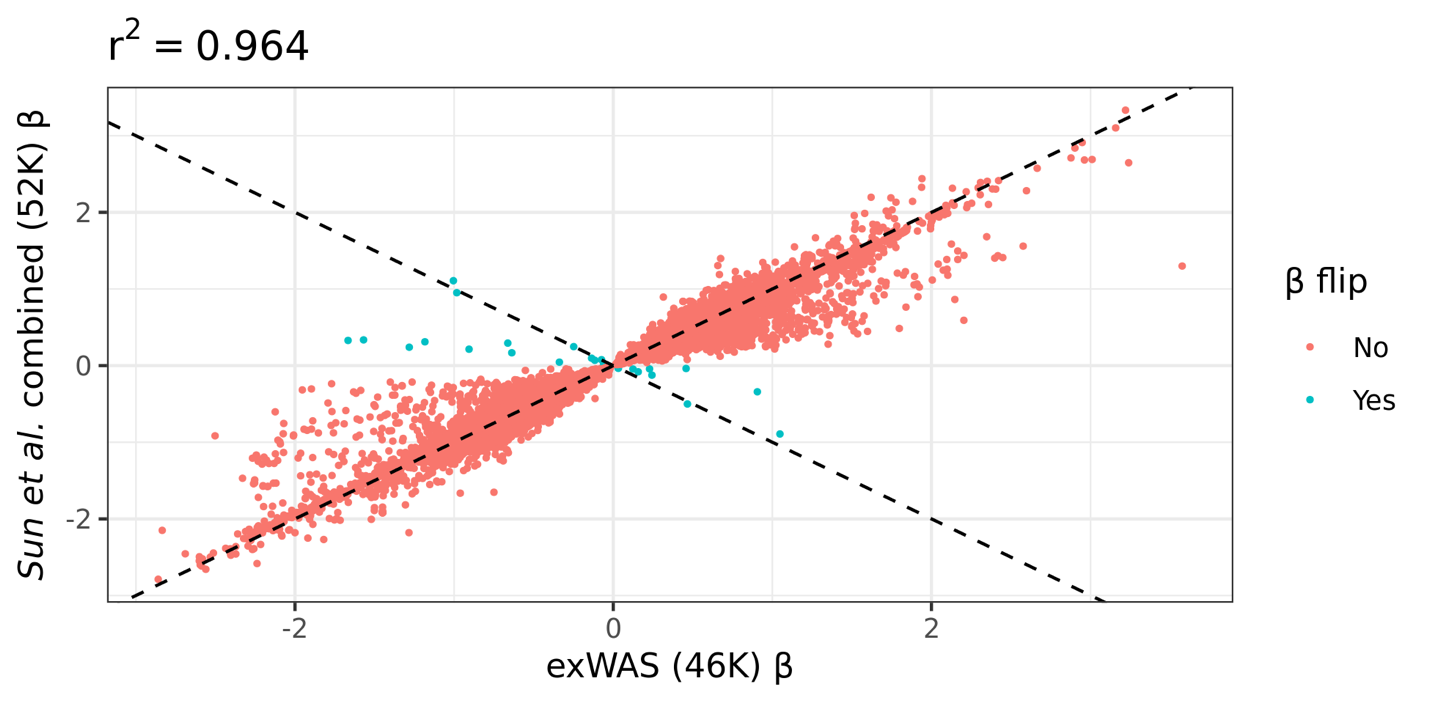


**Correlation of effect sizes between overlapping pQTLs between *Sun et al.* combined and exWAS.** Effect sizes are plotted for variants reaching p<1e-4 in both *Sun et al.*^5^ combined analysis and exWAS (genotypic model). Color indicates whether sign of the effect size is discordant between studies. Dotted lines represent lines of equivalence. The correlation coefficient reflects Pearson’s *r^2^***.**

## Supplementary Figure 3

**
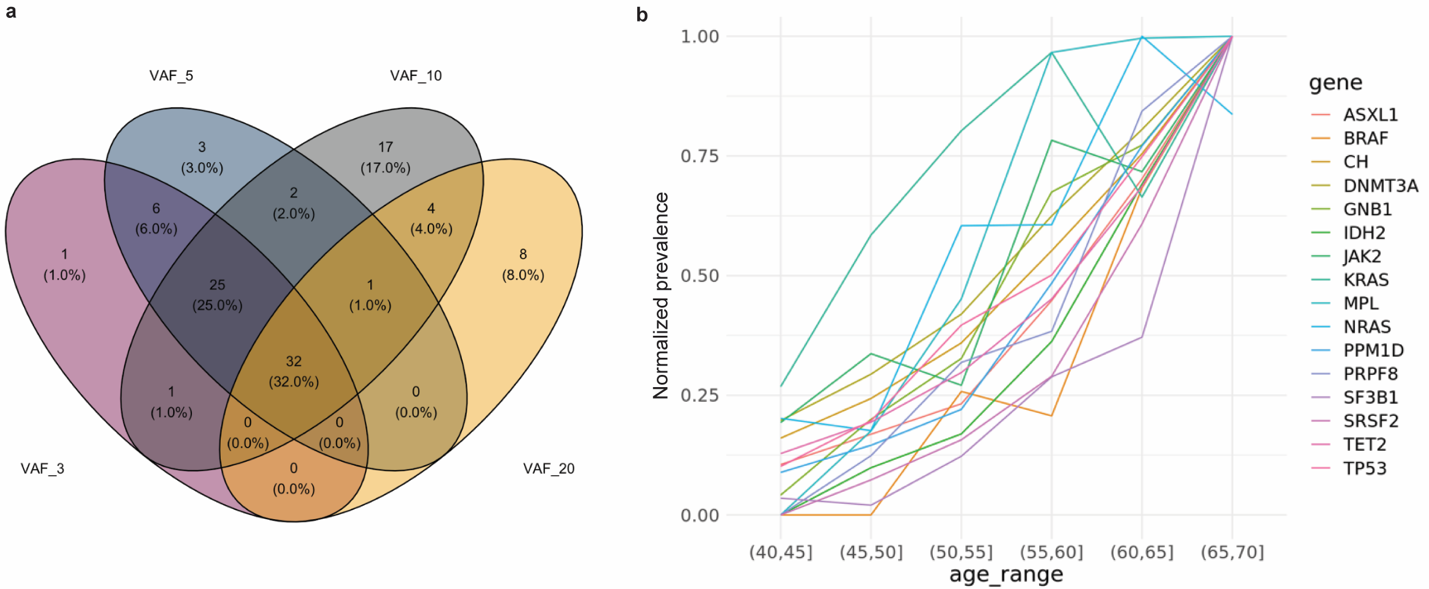
**

**Clonal haematopoiesis. (a)** Venn diagram illustrating the pQTL associations detected at different variant allele frequency (VAF) cut-offs (Methods). **(b)** The prevalence of putative somatic variants by age in 15 genes known to be associated with clonal haematopoiesis and myeloid malignancies. We normalized the prevalence (y-axis) by dividing age by the max prevalence for each gene. “CH” refers to variants observed in all 15 genes.

## Supplementary Figure 4


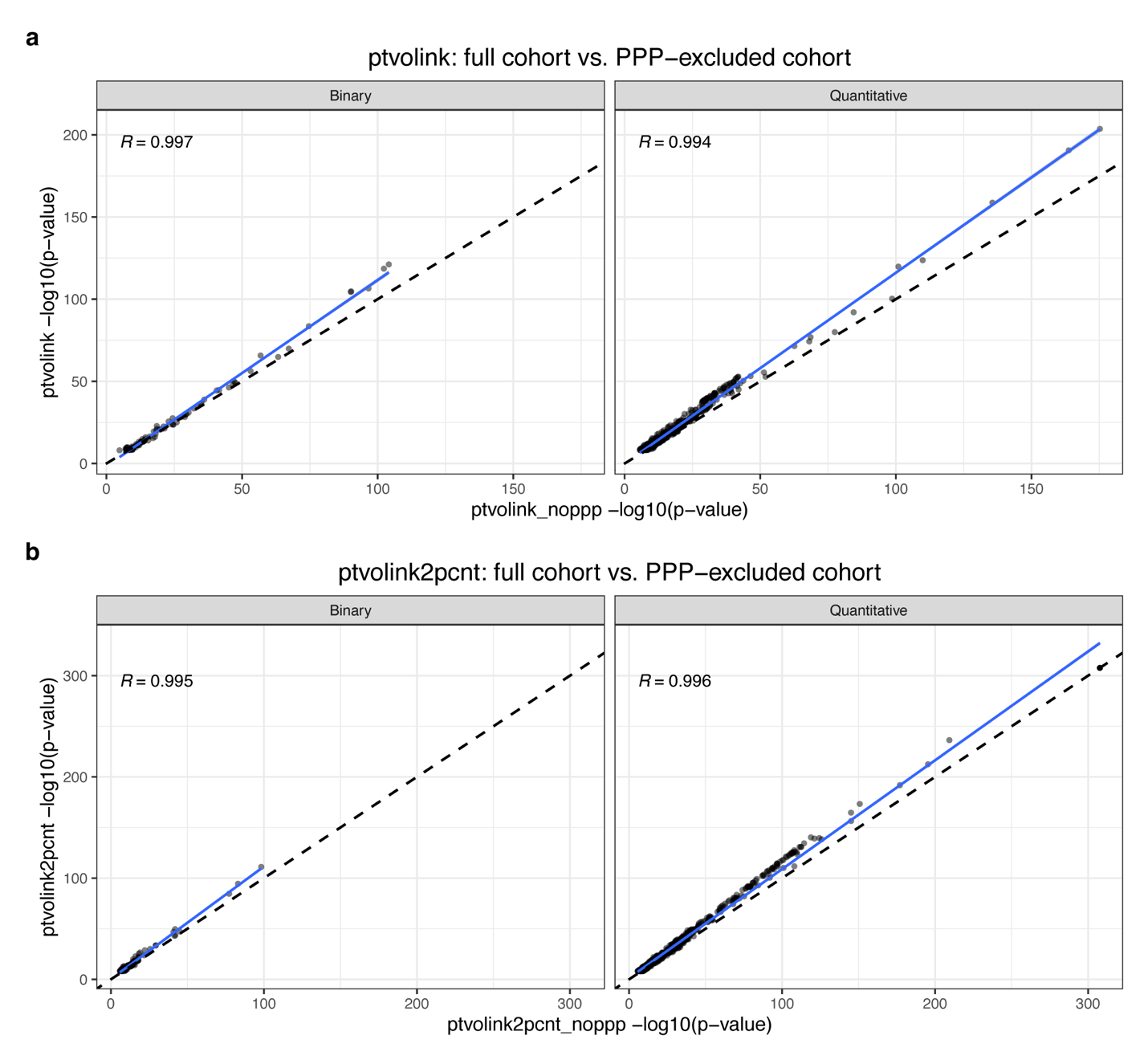


**Concordance of p-values when excluding the UKB-PPP individuals from the pQTL-augmented collapsing analyses.** **(a)** Scatter plot of -log_10_(p-values) from the significant (p≤1x10^-8^) gene-phenotype associations detected in the ptvolink model on the full European UKB cohort (y-axis) versus the ptvolink_noppp model (x-axis), in which we excluded 46,327 individuals who were part of the UKB-PPP cohort. **(b)** Same as (a) but for the ptvolink2pcnt and ptvolink2pcnt_noppp models. The axes are capped at -log_10_(*p*)=305. The R values represent Pearson's correlation coefficient.

## Description of Supplementary Tables

### Supplementary Table 1 – Gene-Protein mapping

Provided as external file. List of the proteins included in the Olink Explore and Olink Expand panels, as well as their gene names (HGNC symbols).

### Supplementary Table 2 – ExWAS (p<1e-7)

Provided as external file. List of ExWAS variants that achieved a p-value<1x10^-7^. P-values were generated via linear regression. Cis_trans_position column refers to whether the pQTL was cis, trans, or “cis-position, trans-gene.” “Pan_p” and “Pan_beta” are the p-value and β values from the pan-ancestry analysis. All other statistics refer to the European-only analysis.

### Supplementary Table 3 – ExWAS and collapsing analysis permutations

Provided as external file. Results from the n-of-1 permutation analysis for the ExWAS and collapsing analysis (Methods). The table also includes results from the empirical null synonymous variant collapsing model. P-values were generated via linear regression.

### Supplementary Table 4 – lambda dist.

Provided as external file. Lambda distributions for the ExWAS and collapsing analyses.

### Supplementary Table 5 – ExWAS tallies

Provided as external file. Number of ExWAS variants per variant effect class. The number of study-wide significant cis-pQTLs versus the total number of tested variants binned by functional category and minor allele frequency (MAF) stratum. For each variant-protein abundance association, we retained the most significant association across the three ExWAS models (i.e., genotypic, dominant, and recessive). The P-values reflect Binomial exact tests (one-sided; alternative hypothesis = greater) comparing the observed versus expected proportion of variants in each cell of the table. Variants were grouped into bins based on the following SnpEff annotations:

PTV – stop gain/loss: stop_gained, start_lost, stop_lost

PTV – frameshift: frameshift_variant, bidirectional_gene_fusion, exon_loss_variant, gene_fusion

PTV – canonical splice: splice_donor_variant, splice_acceptor_variant

Inframe Indels: conservative_inframe_insertion, conservative_inframe_deletion, disruptive_inframe_deletion, disruptive_inframe_insertion

Missense: missense_variant, missense_variant&splice_region_variant

Synonymous: synonymous_variant, synonymous_variant&splice_region_variant, stop_retained_variant, initiator_codon_variant, stop_retained_variant&splice_region_variant

Noncoding: splice_region_variant, non_coding_transcript_exon_variant, 3_prime_UTR_variant, 5_prime_UTR_variant, 5_prime_UTR_premature_start_codon_gain_variant, intragenic_variant,

5_prime_UTR_truncation

### Supplementary Table 6 – Turbidimetric comparisons

Provided as external file. Non-synonymous *cis*-CDS pQTL associations across five proteins that were also assayed via independent turbidimetric assays. The turbidimetric p-values and betas were from an independent ExWAS we performed on ~470,000 UKB participants (Methods). P-values were generated via linear regression.

### Supplementary Table 7 – PCSK9/LDL comparisons

Provided as external file. Non-synonymous *cis*-CDS pQTLs in *PCSK9* and their related associations with low-density lipoprotein (LDL) measured in ~470,000 UKB participants (Methods). P-values were generated via linear regression.

### Supplementary Table 8 – Collapsing pQTLs

Provided as external file. List of gene-level collapsing analysis associations with a p-value < 1x10^-4^. P-values were generated via linear regression. Cis_trans_position column refers to whether the pQTL was cis, trans, or “cis-position, trans-gene.” “Pan_p” and “Pan_beta” are the p-value and β values from the pan-ancestry analysis. All other statistics refer to the European-only analysis.

### Supplementary Table 9 – GNPTAB trans-pQTLs

Provided as external file. *Trans-*pQTLs for *GNPTAB* from the ptv collapsing analysis model (p≤1x10^-8^). We note whether the *trans* protein is a known lysosomal gene in the “Lysosomal?” column. The OMIM gene column indicates whether the protein is known to be associated with a lysosomal storage disease in OMIM. P-values were generated via linear regression.

### Supplementary Table 10 – multi-ancestry collapsing pQTLs

Provided as external file. List of gene-level collapsing analysis associations with a p-value < 1x10^-4^ in the pan-ancestry collapsing analysis models. P-values were generated via linear regression.

### Supplementary Table 11 – Ligand-receptor pairs

Provided as external file. List of *trans-*CDS pQTLs from the collapsing analysis that correspond to ligand-receptor pairs. P-values were generated via linear regression.

### Supplementary Table 12 – CHIP models

Provided as external file. List of qualifying variant models used in the CHIP analysis.

### Supplementary Table 13 – CHIP pQTLs

Provided as external file. List of pQTLs observed in the CHIP collapsing analysis models (p<1x10^-4^). P-values were generated via linear regression.

### Supplementary Table 14 – Studied phenotypes in the PheWAS

Provided as external file. List of binary and quantitative traits included in the phenome-wide association study.

### Supplementary Table 15 – Binary PheWAS

Provided as external file. Results of gene-phenotype associations from the pQTL-informed PheWAS analysis (p<1x10^-4^) for binary phenotypes. P-values were generated via two-tailed Fisher’s exact test.

### Supplementary Table 16 – Quant PheWAS

Provided as external file. Results of gene-phenotype associations from the pQTL-informed PheWAS analysis (p<1x10^-4^) for quantitative traits. P-values were generated via linear regression.

### Supplementary Table 17 – Cautionary variants

Provided as external file. Variant IDs for variants that we observed to have a batch effect (Methods).

### Supplementary Table 18 – Panel concordance

Provided as external file. Concordance of pQTL p-values for proteins measured on multiple Olink panels. P-values were generated via linear regression.

### Supplementary Table 19 – Gene coverage

Provided as external file. Average coverage for each protein-coding gene across all individuals in the cohort.

### Supplementary Table 20 – CHIP variants

Provided as external file. List of pre-defined CHIP variants.

##

## Supplementary References

72 Pietzner, M. *et al.* Synergistic insights into human health from aptamer- and antibody-based proteomic profiling. *Nat Commun* **12**, 6822, doi:10.1038/s41467-021-27164-0 (2021).

73 Katz, D. H. *et al.* Proteomic profiling platforms head to head: Leveraging genetics and clinical traits to compare aptamer- and antibody-based methods. *Sci Adv* **8**, eabm5164, doi:10.1126/sciadv.abm5164 (2022).
